# Is Fitts’ Law Continuous in Discrete Aiming?

**DOI:** 10.1371/journal.pone.0041190

**Published:** 2012-07-18

**Authors:** Rita Sleimen-Malkoun, Jean-Jacques Temprado, Raoul Huys, Viktor Jirsa, Eric Berton

**Affiliations:** 1 UMR 7287 Institut des Sciences du Mouvement E.J. Marey, CNRS and Aix-Marseille Université, Marseille, France; 2 UMR INSERM 1106 Institut des Neurosciences des Systèmes, Faculté de Médecine La Timone, Marseille, France; McMaster University, Canada

## Abstract

The lawful continuous linear relation between movement time and task difficulty (i.e., index of difficulty; *ID*) in a goal-directed rapid aiming task (Fitts’ law) has been recently challenged in reciprocal performance. Specifically, a discontinuity was observed at critical *ID* and was attributed to a transition between two distinct dynamic regimes that occurs with increasing difficulty. In the present paper, we show that such a discontinuity is also present in discrete aiming when *ID* is manipulated via target width (*experiment 1*) but not via target distance (*experiment 2*). Fitts’ law’s discontinuity appears, therefore, to be a suitable indicator of the underlying functional adaptations of the neuro-muscular-skeletal system to task properties/requirements, independently of reciprocal or discrete nature of the task. These findings open new perspectives to the study of dynamic regimes involved in discrete aiming and sensori-motor mechanisms underlying the speed-accuracy trade-off.

## Introduction

How movement kinematics and control mechanisms relate to the speed/accuracy trade-off in target-directed aiming tasks has been a central issue in human movement research for over a century [1]. The simple (linear) model proposed by Fitts [2], later known as Fitts’ law, greatly contributed to the understanding of the general principles underlying the complex relation between environmental constraints and movement kinematics. A central aspect in this respect was the introduction of the index of difficulty (*ID*), which measures the amount of information (in bit, [3]) that needs to be processed to ensure accurate movement ending on a target of a fixed width (*W*) and a fixed distance (*D*). Fitts’ law relates movement time (*MT*) to the index of difficulty following the equation: 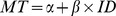, where *α* and *β* are empirical constants depending upon the individual and the task, and 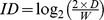.

Fitts’ law has been found to be pertinent to describe both reciprocal and discrete movements [2], [4], and to apply to a large variety of motor tasks (see [5]–[8] for reviews). Thus, it has been widely used as a reference model to investigate sensori-motor processes underlying goal-oriented behavior (e.g., [9]–[12]). Conversely, some scientists have investigated the factors that could alter Fitts’ lawful relationship. Actually, despite its general robustness to predict *MT’s* in a large set of task conditions, Fitts’ law was shown to present some limitations in predicting *MT’s* for low *ID* values (<3 bits) (e.g., [13]–[16]). In order to overcome these limitations and improve the continuous fitting of *MT*, alternative formulations of the speed-accuracy trade-off (e.g., [17]) and *ID* (e.g., [16]) were proposed. More recently, Huys et al. [18] observed a different deviation from Fitts’ law linearity in a reciprocal aiming task. Specifically, a breakpoint was found in the relation between the duration of the acceleration phase (i.e., the first movement portion up to peak velocity) and the (effective) index of difficulty (located at *ID* = 5.88±0.43). In particular, while the duration of the deceleration phase (*DT*, that is, movement duration following peak velocity) continued to increase linearly with increasing *ID*, the duration of the acceleration phase (*AT*) remained approximately unchanged. Since *MT* is a linear addition of *AT* and *DT*, Fitts’s law was considered to be discontinuous in reciprocal aiming. Using the phase spaces’ vector field reconstruction [19]–[21], Huys et al. [18] showed that the discontinuity was concomitant with a transition in operative motor control mechanisms. Specifically, the breakpoint separated two dynamic regimes, one associated with a limit-cycle dynamics operating before the discontinuity, and another associated with a fixed-point dynamics afterwards. These dynamic regimes are typically associated with rhythmic (performed at lower difficulty values) and discrete movements (performed at higher difficulty values), respectively [22], [23].

In that regard, the literature on rhythmic and discrete movements (at least so in the context of Fitts’ task) suffers from some ambiguity due to different conceptions of how to conceive of, define, and identify these movements. One often-used criterion is the harmonicity (*H*) index [24]–[26], which provides a continuum bounded between zero and one. Movements are considered discrete (rhythmic) for *H* <0.5 (*H* >0.5). Consistent with such a continuum, movement as often discussed as being “more” or “less” discrete (or rhythmic). Others, including the present authors, classify movements using principles from dynamical system theory, i.e., by their underlying phase flow topology (cf. [23], [27], [28]) as it allows for unambiguous classification. Accordingly, neither a mapping nor an overlap between classes exists such that ‘more or less’ discrete (rhythmic) makes no sense (even though, obviously, movements can be more or less harmonic). No (trivial) mapping exists between flow topologies and kinematic measures (such as the *H*-index).

Regardless, Huys et al. [18] provided evidence that the transition between dynamic regimes (also) entails a kinematical discontinuity, which was visible through changes in how acceleration and deceleration phases are affected by *ID*. In addition, following the ID/AT breakpoint, Huys et al. [18] found an increase in the variability of movements during target approach with increasing task difficulty. This finding was interpreted as a decrease in the fixed-point’s attractiveness as target width decreased, which in the presence of noise (inherently present in the neuro-behavioral system) results in enhanced variability.

Thus, according to this framework, Fitts’ law discontinuity (in reciprocal aiming) seemingly results from a transition between two dynamic regimes of movement organization, corresponding to fixed point and limit cycle dynamics, presumably reflecting underlying changes in the sensori-motor control mechanisms. Relating this discontinuity to the way the neuro-muscular system dynamically manages the speed-accuracy trade-off in easy and difficult aiming tasks is an appealing hypothesis. If confirmed, it would significantly contribute to the understanding of the general principles governing the sensori-motor control underlying speed-accuracy trade-off. In this perspective, the following step consists in putting Huys et al.’s [18] rationale to the test by using a discrete movement task. According to the aforementioned theoretical framework, we predict that no *ID/AT* discontinuity should be observed, since the transition between limit-cycle and fixed-point dynamic regimes is not relevant in discrete aiming. Conversely, we expect to observe a progressive increase in trajectory variability with *ID*, in particular, for the second portion of the movement, that is, the deceleration phase. In addition, Huys et al. [18] assumed that the discontinuity is related to the level of task difficulty; accordingly we expect that it should be independent of whether *ID* is scaled via *W* (as in Huys et al.’s study) or *D* changes. To test these hypotheses, we first replicated Huys et al.’s [18] experimental design in a discrete aiming task (experiment 1), we then compared the results to the ones observed in the task conditions where *ID* was scaled via changes in target distance (experiment 2).

## Materials and Methods

Two discrete target-directed aiming experiments were carried out. Participants and experimental conditions were comparable to Huys et al.’s [18] study and are detailed in table 1 (additional details on the used experimental conditions are provided in Table S1 for experiment 1 and Table S2 for experiment 2). In the first experiment (experiment 1), task difficulty (*ID*) was manipulated through changes in target size. In the second experiment (experiment 2) *ID* was manipulated through scaling the target distance. Participants held a stylus (18 g, 156.5×14.9 mm, ∼1 mm tip) in their right hand and were required to move across a digitizer (Wacom Intuos4 XL, 1024×768 pixel resolution; 150 Hz sampling frequency of stylus position), from a starting position to a target placed away from their body in the sagital plane. Starting from a home position, they were instructed to reach the target as fast and as accurate as possible by performing a discrete movement (zero velocity at the beginning and the end of the trial). Task conditions were randomly ordered and included 20 repetitions each. If the target was missed for more than two trials, these trials were redone at the end of the block (mean error rates across participants were lower than 5% in all *ID* conditions). All conditions were preceded by 3 unrecorded familiarization trials.

**Table 1:** Participants and experimental conditions details for each experiment.

	Experiment 1	Experiment 2
Number of participants* (mean age in years)	10 (30)	10 (26)
Number of conditions	10	9
ID range in bit	3–7.5 (0.5)	3–7 (≈0.5)
Manipulated parameter (range in cm)	W (5–0.2)	D (2–32)
Fixed parameter (value in cm)	D (20)	W (0.5)

* All participants were self-declared right-handed.

Displacement time series were acquired from the digitizer via custom made software. Fifteen trials over the performed twenty were analyzed in each condition (the first three and the last two were excluded as they were often less consistent with the rest of the trials). Data were smoothed with a dual-pass Butterworth Filter (10 Hz). The “optimal algorithm” of Teasdale et al. [29] was used to determine movement onset and offset on the basis of velocity profiles, obtained by differentiating displacement data. The critical velocity value was obtained by multiplying peak velocity by .04. Movement time (*MT*) was defined as the time between movement onset and offset. Acceleration (*AT*) and deceleration times (*DT*) were defined as the duration prior to and following peak velocity, respectively. Effective target width (*W_e_*) was calculated from the standard deviation of movement end points for each participant in each condition (see [7], [16]). In order to investigate whether participants conformed to the prescribed *ID* or whether a new effective one (*ID_e_*) should be calculated, the effective distribution of end points (centered on the mean amplitude and bounded by the calculated *W_e_*) was compared to the prescribed one (centered on target distance and bounded by the target edges). Since the comparison between the effective and the prescribed performance of the participants yielded no significant statistical difference (t-test, p<.05), the initially fixed *ID’s* were used in the subsequent analyses.

The relation between *ID* and the temporal variables (*MT, AT, DT*) was analyzed using the following linear equation: 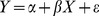; the *α* and *β* terms and their respective Standard Error, were estimated using the simple Ordinary Least Square (*OLS*) estimator. Mean Squared Error (*MSE*) and the coefficient of determination (*R^2^*) were also computed. To study the continuity of the relation between the above-mentioned temporal variables and *ID*, we first used a non-parametric smoothing spline fitting (with the smoothing parameter “*p*” set at.99), which enabled us to visually detect and localize a breakpoint in the linear relations. Then a piecewise linear regression model with a combination of two linear relations was computed following the equation: 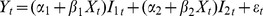; with: *I*_1*t*_  = 1 for t  = 1;…;j otherwise 0; and *I*_2*t*_  = 1 for t  =  j+1;…;k otherwise 0; j  =  number of observations before the breakpoint, where k+j equals the total number of observations. To confirm the presence of the breakpoint(s), we tested if the slopes were significantly different in between the equations (Student’s t statistic, H0: *β*_1_ =  *β*_2_ at 95% confidence interval). Once the piecewise model was validated, its *R^2^* and *MSE* values were compared to those of the simple linear regression model. The best model to fit the data was the one having the smallest *MSE* and the highest *R^2^*. The regression analyses were primarily applied to each participant’s data before applying it to mean participants’ data. This procedure aimed to ensure the consistency between the reported data across participants and the ones observed at an individual level.

Principal component analysis (PCA) was used to study the movement trajectory variability (see [18], [30] for details on the use of PCA to study variability) prior to and after peak velocity that is, during the acceleration and deceleration phases, respectively. PCA is generally used to reduce the dimensionality of data sets by extracting the smallest number components (modes) that account for most of the variation in the original data without losing significant information. The eigenvalue of each mode reflects the amount of variance captured by it so that examination of the first mode’s eigenvalue can be used to assess the degree of variability in the data (especially if it is close to one). Accordingly, the eigenvalue of the first mode was used as a measure of the inter-trial trajectory variability for each movement phase. It was computed for each participant in all conditions. Then mean across-participant values were calculated for each *ID* condition. The eigenvalues of both acceleration and deceleration phases were subjected to repeated-measures ANOVA with *ID* as a within participant factor. The level of significance was set at 5%.

Informed written consent was obtained from every participant prior to testing.

## Results

### (a) Experiment 1

**Figure 1:**
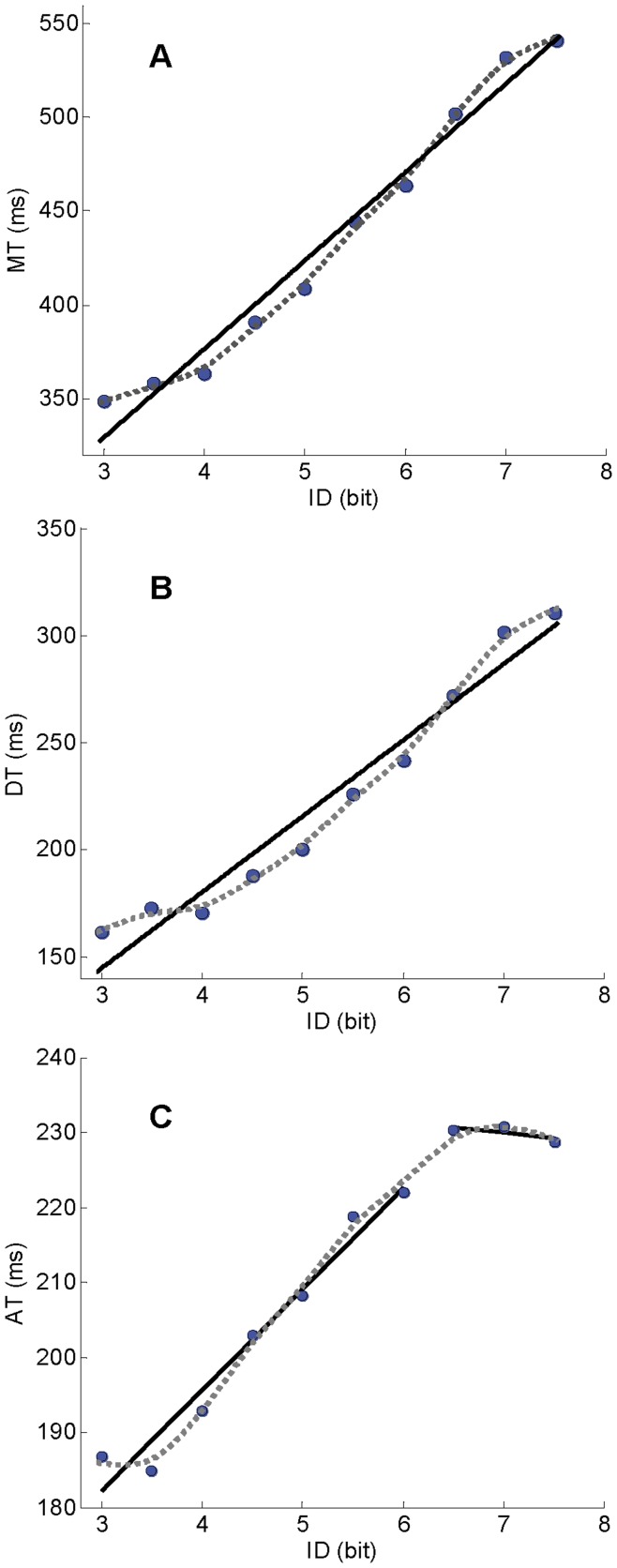
Data and fittings for experiment 1. (A) movement time; (B) deceleration time; and (C) acceleration time. Each dot represents mean value across participants for the corresponding *ID* condition. Dashed grey lines represent spline fitting curves. Black lines represent linear regressions.

**Figure 2:**
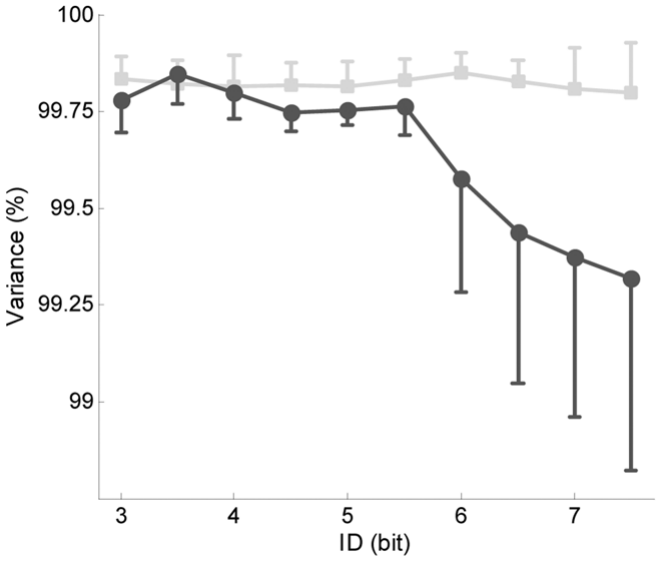
PCA analysis results. Variance captured by the first mode for *AT* (square markers, light grey line) and *DT* (round markers, dark grey line).

The statistics of the simple linear regression analysis for *MT*, *AT* and *DT* are presented in table 2. Though all three variables presented an increasing linear relation with *ID*, Fitts’ law equation fitted better *MT* and *DT* data than *AT* data. The non-parametric spline fitting showed no significant discontinuity in the *ID/MT* and *ID/DT* relations for the investigated range of *ID* (figure 1). It revealed however a discontinuity in the *ID/AT* relation (figure 1) with a curvature around 6.5 bits (±0.5 bits between-subjects variability). The presence of a breakpoint at 6.5 bits was confirmed by the piecewise regression analysis (figure 1). The double regression model (*α_1_* = 141.70, *p*<.05; *β_1_* = 13.48, *p*<.05; *α_2_* = 240.58, *p*<.05; *β_2_* = −1.52, *p*>.05) presented a better fit than the simple linear model (*MSE* = 11.91, *R^2^* = 0.99). The PCA analysis, performed on *AT* and *DT* data (figure 2), showed an increase in *DT* variance after 5.5±0.5 bits (*F*(9, 63) = 7.31, *p*<.05), while no significant change occurred in *AT*’s (*F*(9, 63) = .42190, *p*>.05).

**Table 2:** Linear regressions’ estimates for mean data across participants.

	Experiment 1			Experiment 2		
	*MT*	*AT*	*DT*	*MT*	*AT*	*DT*
**R^2^**	.97	.94	0.95	0.98	0.98	0.98
**MSE**	148.84	21.49	154.75	448.17	79.37	211.28
**α**	186.93	149.94	37.77	−191	−66	−124
**β**	47.30	11.56	35.5	115.5	47.96	67.79

All coefficients were significant at 5% level.

### (b) Experiment 2

**Figure 3:**
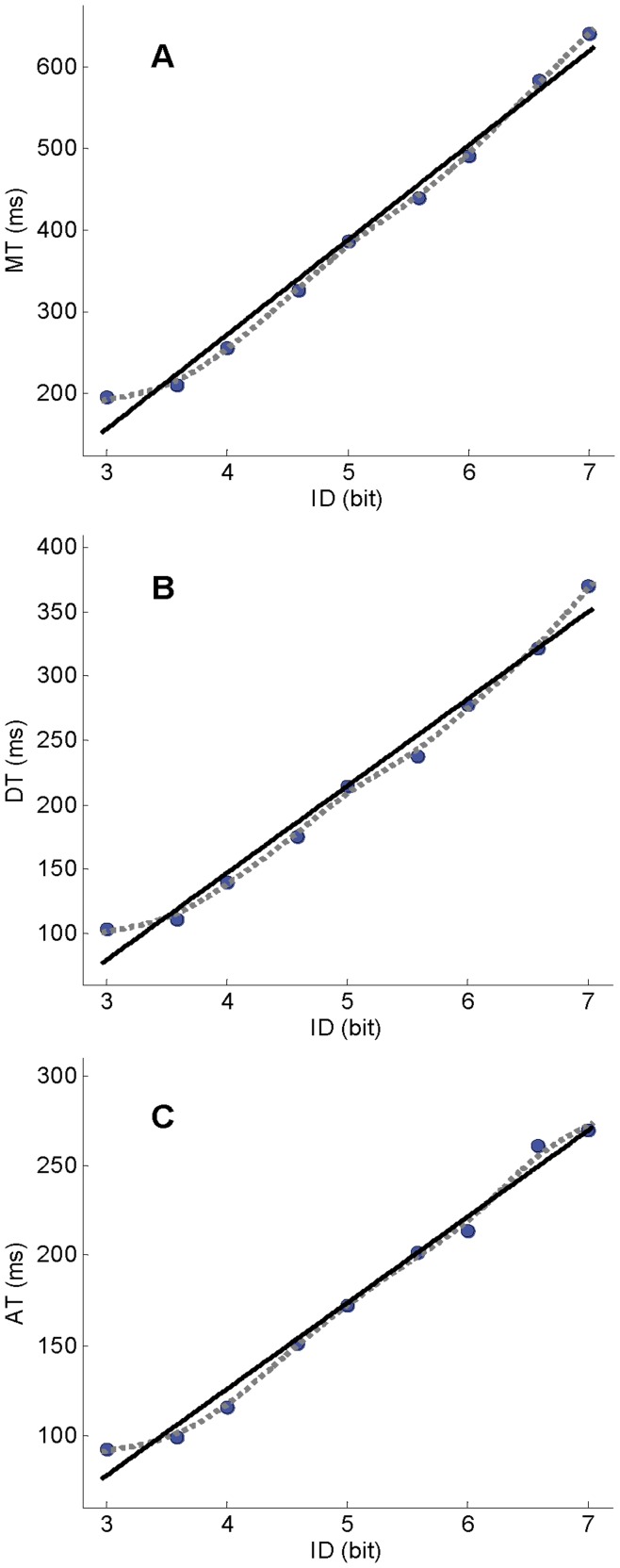
Data and fittings for experiment 2. (A) Movement time; (B) deceleration time; and (C) acceleration time. Each dot represents mean value across participants for the corresponding *ID* condition. Dashed grey lines represent spline fitting curves. Black lines represent linear regressions.

Fitts’ law linear equation fitted the *MT*, *AT* and *DT* data well (table 2). The linear relation between *ID* and the studied temporal variables was clearly visible on the non-parametric fitting curve (figure 3). However, a slight upward curvature was observed for very low *ID* values. Since no discontinuity close to the one reported by Huys et al. [18] was observed, no further analyses were performed.

## Discussion

The goal of the present work was to determine whether Fitts’ law discontinuity is exclusively related to a transition from limit-cycle to the fixed-point regime, which would make it limited to reciprocal aiming as it was recently hypothesized by Huys et al. [18].

The results observed in the first experiment using a discrete rapid-aiming task in which only target size was manipulated were, unexpectedly, similar to those reported by Huys et al. [18] for reciprocal aiming. Specifically, we observed a discontinuity in the *ID/AT* relation for high difficulty levels, which did not result in an observable discontinuity in the *ID/MT* relation, but was concomitant to an increase in the variability of movement trajectory during target approach. The fact that we failed to identify a discontinuity in *MT* probably reflects that it is harder to detect; after all, *DT*, which scales linearly with *ID*, accounts for most of the variance in *ID*-*MT* space. Regardless, given *MT*  =  *AT* + *DT*, we interpret our results as indicative of a discontinuity in Fitts’ law (see also above), but are aware this interpretation based on the *MT*’s components may evoke debate.

The common findings observed in Huys et al’s study [18] and our first experiment demonstrate that cyclic and discrete Fitts’ tasks share comparable behavioral discontinuities. The transition from a limit-cycle to a fixed-point regime cannot be responsible of the discontinuity that was observed in discrete aiming. Indeed, since in the discrete task participants have to stop their movement when they reach the target, a fixed-point is necessarily implicated. Therefore, if the observed discontinuities share a common origin, it should be owed to a more general mechanism underlying neuro-behavioral adaptation to the task difficulty, which triggers a transition in the operating dynamic regime. The question remains on what kind of (other than a fixed-point) dynamics could be implicated in discrete movement control. In that regard, theoretically, discrete movements can be generated by (at least) three distinct dynamic mechanisms. In one case, a trajectory traverses a phase space between two co-existing fixed points (bi-stability); in this case the phase flow is invariant on the time scale of the movement (e.g., [28]). In both other cases, the phase flow changes during movement execution. Schöner’s framework [27] provides one such scenario; accordingly, the system is at a fixed point, which vanishes to make way for a limit cycle, and the movement unfolds. Shortly before movement termination, the limit cycle disappears and another fixed point emerges to which the system comes to a stop. Alternatively, a fixed point may traverse the phase space and consequently change its structure (the flow) on the time scale of the movement (this mechanism, can be seen as a dynamical view of equilibrium point hypotheses; see [31]–[33] for equilibrium point control, and [23], [34], [35] for motivations of dynamical formulations thereof). By hypothesis, this latter mechanism may underlie the (visually-guided) approach to small targets and is thus a possible candidate for the regime operating after the breakpoint. This proposition, at least its dynamical formulation, remains hypothetical for the time being, but it certainly deserves to be further investigated, since it permits to formalize the link between the kinematic adaptations and the dynamic transition mechanisms underlying the speed-accuracy trade-off control in both cyclic and discrete movement tasks. Before the breakpoint, both Schöner’s system as well as the bi-stable one could account for the movement generation. We deem it unlikely however, that Schöner’s view can account for movement generation across the entire range of *ID*s as it is not consistent with the presence of a breakpoint.

The fact that, in both reciprocal [18] and discrete aiming tasks (experiment 1), the breakpoint was located around 6 bits (5.88 and 6.5, respectively) might suggest that the discontinuity in Fitts’ law primarily depends on the *ID* level. This hypothesis was investigated in experiment 2 by using an *ID* range that was comparable to the one used in experiment 1 but obtained by increasing the target distance. Compared to the *W* manipulation (experiment1), the *D* scaling (experiment 2) had a stronger effect on movement duration, with a steeper slope of *ID/MT* function (table 2), but induced an equivalent lengthening in the duration of acceleration and deceleration movement phases (table 2). More importantly, *ID* presented a close-to-perfect linear relation with all temporal variables (*MT*, *AT*, *DT*), and no abrupt breakdown was observed in the contribution of movement phases in the achievement of the task across difficulty levels. These findings show that kinematic discontinuities do not depend upon the level of difficulty per se (which may also be the case for the transition between dynamic regimes). Rather, it seems to be related to accuracy constraints (i.e., *W* manipulation). Previous findings reported in the reciprocal aiming literature support this hypothesis. Indeed, the switch from cyclical to discrete motion with increasing difficulty has been principally observed in movement tasks where *ID* was manipulated via target width (e.g., [11], [22], [24], [25], [36]). Here, we show that the *ID*/*AT* discontinuity is specific to a class of Fitts’ tasks in which movement accuracy constraints constitute the key variable to increment the *ID*.

The observed differences between *W*- and *D*-induced adaptations confirm the specificity of the effects of the constraints arising from task properties on the speed-accuracy trade-off, movement kinematics, and ultimately, transitions between modes of movement control. Thus, the present results might bring the interpretation back to the task-dynamic approach that was introduced by Saltzman & Kelso [37] and, more recently, extended by Bootsma et al. [38] to account for the continuous coupling between perceptual and motor processes and its consequences on kinematic and dynamic adaptations. In the present context, the interest of this approach is that it is not formally restricted to adaptations observed in cyclic movement tasks. Indeed, in this theoretical framework, regardless of the nature of the task, both global *MT’s* and the observed *ID/AT* discontinuity can be considered as emergent features of the continuous interplay between mechanical (movement-related) and informational (perception-related) flows/constraints. From the preception-action coupling perspective, the observed abrupt change in how *ID* affects movement organization (i.e., the duration of acceleration and deceleration phases) might be triggered by a permanent property of Fitts’ task-space (i.e., the space where the relationship between the movement and the target is controlled e.g., [39]). Such property would presumably relates to the relation between the ongoing motion and target width (i.e., accuracy constraint). Accordingly, to ensure an optimal speed-accuracy trade-off, a specific spatio-temporal relationship between the offset/onset of acceleration and deceleration movement phases is needed. One could suggest that this relationship (i.e., the contribution of each of these two phases) emerges from the continuous coupling between the movement and an informational variable generated on-line by the approach of the target (see [40] for a similar reasoning in coordination between reaching and grasping). As a result of this coupling, for a given target distance, there might be a threshold in accuracy constraints (critical *W*) beyond which the movement organization abruptly changes so that the speed-accuracy trade-off would be exclusively managed by modulating the deceleration time. The pertinence and the exact nature of the informational property controlling the contribution of movement phases, as well as how it may shape the self-assembling of the underlying dynamics (oscillatory or otherwise) within the neuro-behavioral system, remains to be identified. This could be done using the same experimental strategy as Fernandez and Bootsma [39]. We note however that, in this perspective, the account of the presumed underlying dynamic mechanism that would sub-serve the kinematic adaptations to task properties (see [26]) is different from the one suggested by Huys et al. [18]. Particularly, kinematic adaptations are considered to result from the gradual tuning of the (stiffness and damping) parameters of the oscillatory dynamics. Such account seems to be counterintuitive with regard to the increase in the variability of movement trajectories with *ID* and the presence of a kinematic discontinuity.

Whereas most studies focused on the differences between cyclical and discrete movements, our results showed the presence of an abrupt switching in the kinematic strategy adopted by the neuro-musculo-skeletal system to accommodate higher levels of accuracy constraints, regardless of the rhythmic or discrete nature of the aiming task. These findings hint at the presence of a common mechanism to (severe) precision constraints. In this respect, they are of conceptual importance for understanding the speed-accuracy trade-off in a unifying perspective while respecting the distinction between rhythmic and discrete movements. Therefore, though it is not the point of the present issue at stake, in a way, the present study lends credence to previous modeling work that attempted to bridge the gap between cyclic and discrete dynamics (e.g., [27], [28], [41]).

The present study showed that, although Fitts’ task remains one of the most robust and widely used paradigms in motor control literature, it has certainly not yet revealed all its secrets. Such statement should encourage researchers to further investigate on the kinematic adaptations of the speed-accuracy trade-off that are common to both discrete and cyclical aiming tasks under both *W*-induced and *D*-induced *ID* scaling. These adaptations could also constitute a new window into the study of sensori-motor alterations resulting from aging and neuro-degenerative diseases.

This study was approved by the ethics committee of Aix-Marseille University and it conforms to the provisions of the Declaration of Helsinki.

## Supporting Information

Table S1 **Details of the experimental conditions used in experiment 1.**
(DOC)

Table S2 **Details of the experimental conditions used in experiment 2.**
(DOC)
