# Supplementary material for: Is Fitts’ Law Continuous in Discrete Aiming?
Source: PLoS One. 2012 Jul 18;7(7):e41190. doi: 10.1371/journal.pone.0041190 (PMC3399829; doi:10.1371/journal.pone.0041190)
Supplement: Table S1 — Details of the experimental conditions used in experiment 1. (DOC) [file pone.0041190.s001.doc]

| **Condition Number** | **Width (cm)** | **Distance (cm)** | ***ID* (bits)** |
| --- | --- | --- | --- |
| 1 | 5.00 | 20.00 | 3.00 |
| 2 | 3.54 | 20.00 | 3.50 |
| 3 | 2.50 | 20.00 | 4.00 |
| 4 | 1.77 | 20.00 | 4.50 |
| 5 | 1.25 | 20.00 | 5.00 |
| 6 | 0.88 | 20.00 | 5.50 |
| 7 | 0.63 | 20.00 | 6.00 |
| 8 | 0.44 | 20.00 | 6.50 |
| 9 | 0.31 | 20.00 | 7.00 |
| 10 | 0.22 | 20.00 | 7.50 |
